# Dietary Calcium Intake, Vitamin D Status, and Bone Health in Postmenopausal Women in Rural Pakistan

**DOI:** 10.3329/jhpn.v29i5.8900

**Published:** 2011-10

**Authors:** Nicola M. Lowe, Basma Ellahi, Qudsia Bano, Sonia Ali Bangash, Soma R. Mitra, Mukhtiar Zaman

**Affiliations:** ^1^International Institute of Nutritional Sciences and Applied Food Safety Studies, University of Central Lancashire, Preston, United Kingdom; ^2^Department of Clinical Sciences, University of Chester, Chester, United Kingdom; ^3^Emergency Satellite Hospital, Nahaqi, Khyber Pakhtunkhawa, Pakistan; ^4^Khyber Teaching Hospital, Peshawar, Khyber Pakhtunkhawa, Pakistan

**Keywords:** Bone health, Bone mineral density, Calcium, Osteoporosis, Quantitative ultrasound index, 25(OH)D, Vitamin D deficiency, Pakistan

## Abstract

The high prevalence of osteoporosis in Pakistan is of public-health concern. However, there is a paucity of information regarding nutrition and bone density in rural communities. The purpose of this study was to evaluate the dietary and lifestyle factors that impact bone health in Nahaqi. Data were collected from 140 postmenopausal women using an interviewer-administered 24-hour dietary recall questionnaire. Bone mineral density was estimated using the quantitative ultrasound index (QUI). Serum 25(OH)D was measured in fasting blood samples. The QUI scores revealed that 42% and 29% of the women had T-scores, indicative of osteopaenia and osteoporosis respectively. The mean calcium intake was 346 mg/d, which is less than 50% of the recommended daily intake. The QUI correlated with 25(OH)D after controlling for age (p=0.021, r=0.41, r^2^=0.168). Vitamin D deficiency and low intake of dietary calcium are two key factors contributing to poor bone health in this population.

## INTRODUCTION

The prevalence of osteoporosis in Pakistan is high, with 97% of women aged 75-84 years and 55% of women aged 45-54 years predisposed to osteoporosis (1). Such women can present with back pain, loss of height, and stooped posture before fracture occurs. The symptoms are primarily a consequence of reduced bone mineral density and microarchitecture that occurs postmenopause due to a fall in oestrogen levels. Developing peak bone mass is influenced by genetics and multiple lifestyle characteristics (2). Some risk factors for osteoporosis, such as age, maternal history of hip fracture, current smoking, alcohol intake, low body-weight, and falls, are partially independent of bone mineral density (3), and assessment of clinical risk factors is used as a selection method for referral for dual energy x-ray absorptiometry (DEXA). DEXA remains the ‘gold standard’ diagnostic tool for osteoporosis but high cost and low availability restrict its use in primary care in Pakistan. Quantitative ultrasound measurements of the calcaneal bone are also recognized as a potential indicator of the risk of fracture (4). Studies have demonstrated a high correlation between the ultrasound measurements and the bone mineral density at the calcaneus and suggest that for every 1 standard deviation (SD) decrease in broadband ultrasound attenuation (BUA), there is a 2.3-fold increase in the risk of hip fracture (5,6).

Important modifiable factors involved in the optimization of bone mineral density include adequate dietary calcium intake and vitamin D status, and lifestyle factors, including activity levels, parity, and age of menarche and menopause. Calcium-balance studies have concluded that low dietary calcium and/or low absorption of calcium may be a major risk factor for the development of osteoporosis (7,8). However, studies using bone densitometry methods to investigate the role of calcium in the development of osteoporosis have yielded conflicting results (9,10). Vitamin D plays a key role in calcium absorption and homeostasis, and there is increasing evidence supporting the role of vitamin D supplementation in the prevention of falls and fractures in elderly postmenopausal women (11-13). Several studies in Europe have demonstrated that poor vitamin D status is a particular problem in European South Asian immigrant populations. It has been suggested that South Asian diets, coupled with reduced exposure to sunlight, may compromise calcium and vitamin D status (14,15).

In a survey of 608 women aged 40 years or older attending two teaching hospitals in Peshawar, based on calcaneus BUA measurements, 12% of women were osteoporotic, and 35.6% were osteopaenic (16). The number of women with BUA scores, indicative of osteoporosis, increased with age; however, this study did not investigate diet, vitamin D, or lifestyle factors.

There is a paucity of detailed data regarding diet, vitamin D status, and bone health in rural populations in Pakistan, particularly from conservative Muslim regions where the wearing of a full burqa is a common practice and may impact on exposure to sunlight and, thus, vitamin D status.

The purpose of this study was to undertake an indepth investigation of bone health using ultrasound and the dietary and lifestyle factors that influence the achievement and maintenance of optimal bone mineral density in postmenopausal women living in a rural population in Nahaqi, near Peshawar in Khyber Pakhtunkhawa (formerly North West Frontier Province), Pakistan.

## MATERIALS AND METHODS

### Study participants

The study participants were women aged 40-65 years and at least one year postmenopausal. They were identified from the Nahaqi Research Database Project. The women were all resident in union councils—Khazana and Nahaqi—in close proximity to the Emergency Satellite Hospital. These communities are serviced by a team of Lady Health Workers (LHWs), each of whom is responsible for a cluster which is a defined number of households with population of no more than 1,000 individuals in a defined geographical area. Ten women from 14 LHW clusters were invited to participate in the study. The exclusion criteria included use of steroids, calcium and/or vitamin D supplementation, hormone-replacement therapy, renal diseases, diuretic use, and gastrointestinal disorders. All the selected participants fulfilled the criteria, and none was excluded.

### Analysis of diet and lifestyle

The participants were invited to attend the Emergency Satellite Hospital in groups of 10 along with their LHWs. Briefing was given as a group, and the consent and criteria were verified. At the start of the study, each participant completed a health and lifestyle questionnaire that included questions regarding factors known to influence bone health. These included age of menopause and menarche, number of pregnancies, number of children, history of personal fracture and of siblings, smoking, drug-use, occupation, and household income. In addition, food intake was recorded using an interviewer-administered 24-hour recall questionnaire. The questionnaire was administered through face-to-face interview individually, and the researcher recorded the responses. Ranges of cooking utensils (cups, spoons, plates) were used for estimating the portion size. The intake of nutrients was calculated using food tables (17) and an electronic database (Nutrisurvey for windows. Copyright©2007. Dr. Juergen Erhardt Sameo-Tropmed Rcc, Indonesia.www.nutrisurvey.de). This food intake questionnaire was conducted at each subsequent visit to the Emergency Satellite Hospital for the purposes of this study, which occurred on two or three separate occasions in each season.

*Physical activity levels*: Physical activity was also documented at the same time as the diet recall questionnaire was administered. This was done through face-to-face interview individually. The researcher recorded the responses. Each participant was asked to recall their activities for the whole 24 hours, starting from the early morning prayer (waking up) to the next morning prayer. The total amount of physical activity was quantified by multiplying time spent on activities with respective metabolic equivalent (MET) values (18). The total MET-hours (per day) was divided by 24 (total hours in a day) to calculate the physical activity level (PAL) for each participant.

### Bone ultrasound and anthropometry

At the start of the study, height and weight of the participant were recorded and were used for calculating body mass index (BMI). Bone health was evaluated and estimated using BUA and speed of sound measurements of the calcaneus, combined to give the quantitative ultrasound index (QUI) (SAHARA; Hologic Inc., Bedford, MA, USA). The trained technicians performed the test. The results are reported in T-score which is a measure of the bone density of the participants compared to a normal healthy young adult of the same sex. According to the manufacturer of the instrument, a T-score above −1 indicates that the bone density is normal, a score between −1 and −2.4 is indicative of osteopaenia, and a score of −2.5 and below is indicative of osteoporosis.

### Blood 25(OH)D and parathyroid hormone

Fasting blood samples were taken from a representative subgroup of the study women. The subgroup comprised 31 women randomly selected from each of the three categories based on their QUI scores: 13 women with a score above −1, 13 women with a score between −1 and −2.5, and 11 women with a score below −2.5.

Parathyroid hormone (PTH) levels were measured using the chemiluminescent technique (immulite-100 Siemens) and 25(OH)D using the electro-chemiluminescent technique (E-170 Roche) at the Clinical Laboratories of the Aga Khan University Hospital, Karachi. For quality assurance, PTH and 25(OH)D internal control samples, provided by the respective assay manufacturers, were run with every batch of samples from patients. All control values fell within ±2 standard deviation of the reference. In addition, an external control for PTH assay was run by the laboratory on a bi-weekly basis.

### Analysis of data

Statistical tests were performed using the SPSS software for Windows (version 17.0). Repeated measures ANOVA was used for determining the effect of season on the nutrient intake and physical activity level. Pearson's correlation test was used for correlation analysis. A value of p≤0.05 was considered significant.

### Ethical approval

The women gave verbal consent (100% of them were uneducated) and agreed to come for face-to-face interview to the Emergency Satellite Hospital in Nahaqi on at least eight occasions in the 12-month study period. The Faculty of Science Ethics Committee of the University of Central Lancashire approved the study. The study was conducted in accordance with the ethical standards of the University, and the procedures followed were in accordance with the Helsinki Declaration.

## RESULTS

### Health and lifestyle characteristics

Factors that may influence bone health are presented in Table 1. The average BMI was in the overweight range at 27.9 kg/m^2^. However, 5% of the women were underweight (BMI ≤19 kg/m^2^), 31% were in the healthy range (BMI >19≤25 kg/m^2^), 38.1% were overweight (BMI >25≤30 kg/m^2^), and 25.9% were in the obese category (BMI >30 kg/m^2^). None of the women reported ever having used tobacco (cigarette, snuff, or huqqa) or opium. Of the 140 women, 10 reported long-standing illness, including diabetes (n=7) and hypertension (n=2), and one was blind. None reported having sustained a bone fracture herself, nor having a parent or sibling who had sustained a bone fracture. Most women were married (n=92), or widowed (n=46), with one single and one divorced. Their reported occupations included: housewifery (n=29), domestic help (n=44), and other (including midwifery, basket-making, hawking, tailoring) (n=50), and none as retired (n=17). The number of women reporting an average monthly household income in the following ranges were: Rs >30-<300 (n=30), Rs >300-<600 (n=72), Rs >600-<900 (n=20), and Rs >900 (n=18) (US $1=Rs 85.64). None reported having sustained a fracture herself, nor having a sibling who had sustained a fracture. None had undergone a hysterectomy, and none reported ever having used tobacco or recreational drugs.

**Table 1.** Characteristics of participants (n=140)

Characteristics	Mean±SD	Range
Age (years)	52±6	41-67
Weight (kg)	63±13	35-97
Height (m)	1.52±0.13	1.20-1.76
BMI (kg/m ^2^)	27.3±5.7	16.5-48.0
Parity	5±3	0-13
Age (years) of menarche	13±1	12-14
Age (years) of onset of menopause	46±2	40-49
No. of pregnancies	8±4	0-20

BMI=Body mass index;

SD=Standard deviation

### Analysis of diet

The range of foods consumed by the participants was limited. The most commonly-consumed foods are shown in Table 2. Analysis of the nutrient content of the diet and physical activity by season is shown in Table 3. The total energy intake increased significantly with each season from summer to autumn and winter, with a concurrent fall in the percentage of calories coming from carbohydrate, and an increase in percentage of calories from protein during the winter months. This was associated with an increase in calcium and zinc intakes from summer to autumn and autumn to winter. The mean calcium intake was significantly lower than the World Health Organization's dietary recommended intake of 1,300 mg/day, and the more conservative UK reference nutrient intake value for calcium of 700 mg per day (p<0.001). PALs for this group of women were consistent with sedentary, inactive lifestyles (19). There were no significant seasonal changes in PAL.

**Table 2.** Commonly-used foods by participants

Food-group	Commonly-used foods
Milk and milk products	Milk and curd
Cereals and pulses	Wheat, rice, lentil, cow pea, and kidney bean
Carbohydrates	Sugar (sucrose)
Lipids	Fats (ghee)
Meat-group	Meat and poultry
Vegetables	Potato, cabbage, green pea, lady finger, guard, bitter gourd, brinjal, onion, and tomato
Fruits	Use of fruits was very rare

**Table 3.** Physical activity level and daily nutrient intake by season

Outcome measure	Summer (June-August)		Autumn (September-November)		Winter (December-February)	
	Mean	SD	Mean	SD	Mean	SD
No.	140		109		100	
Physical activity level	1.26	0.12	1.28	0.12	1.28	0.12
Energy intake (kcal)	1,646.6 ^a^	341	1,789.2 ^b^	250.4	1,909.5 ^c^	203.4
% of energy from Carbohydrate	61.9 ^a^	6.1	59.9 ^b^	5.2	57.3 ^c^	4.4
Protein	11.4 ^a^	1.3	11.4 ^a^	1.4	14.0 ^c^	2.4
Fat	26.2	5.7	27.3	4.4	27.1	2.6
Calcium (mg)	316.9 ^a^	99.4	354.7 ^b^	128.4	367.1 ^c^	60.0
Zinc (mg)	10.1 ^a^	2.1	11.9 ^b^	1.7	13.9 ^c^	4.4

Values with different superscripts are significantly different from each other according to the season.p<0.05;

Repeated measures ANOVA;

ANOVA=Analysis of variance;

SD=Standard deviation

### Quantitative ultrasound measurements, 25(OH)D, and parathyroid hormone

Quantitative ultrasound measurements were made in 107 of the 140 women recruited onto the study. The T-scores revealed that 32 (30%) of the participants had T-scores within the normal range, 46 (43%) were in the osteopaenic range, and 29 (27 %) had T-scores, indicative of osteoporosis. The median value within each of these three categories are shown in Table 4, along with serum 25(OH)D, and PTH levels were measured in a sample of the participants from each T-score category.

### Analysis of correlation

**Fig. 1.**
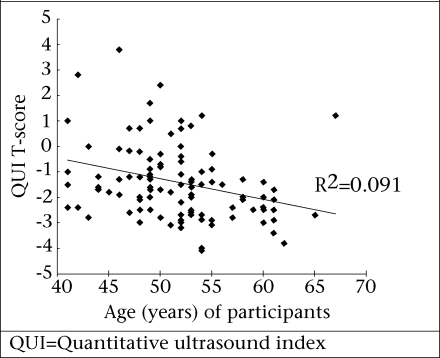
Correlation between QUI T-score and age of participants

**Fig. 2.**
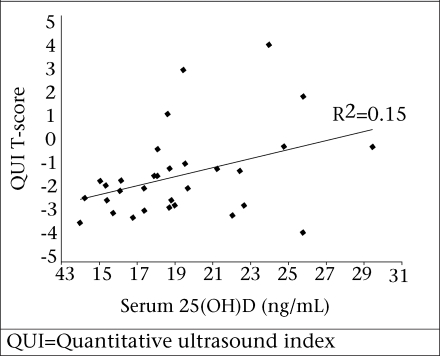
Correlation between QUI T-score and serum 25(OH)D

There was a significant correlation between the QUI T-score and age (p=0.001, r=0.303, r^2^=0.091) and between the QUI score and serum 25(OH)D (p=0.031, r=0.387, r^2^=0.149). These are shown in Figure 1 and 2 respectively. When removing the effect of age, serum 25(OH)D_3_ still accounted for 16.8% of the variation in the QUI (p=0.021, r=0.41, r^2^=0.168). There were no significant correlations between the QUI and the BMI, PTH, calcium intake, number of children, age of menarche or menopause, protein intake, or physical activity levels.

## DISCUSSION

The purpose of the study was to explore the prevalence of osteoporosis and osteopaenia and the dietary and lifestyle factors that may affect bone health in a relatively-small sample of postmenopausal women living in a region near Peshawar in Khyber Pakhtunkhawa. This rural region is served by the Emergency Satellite Hospital in Nahaqi, and the population here is uneducated and of lowsocioeconomic status. Over 70% of the participants reported household incomes of less than Rs 600 per month. The poverty-line is considered to be Rs 723 per month. It was hypothesized that poor diet (primarily a result of poverty) and suboptimal vitamin D status, due to the wearing of a full burqa, thus, limiting exposure to sunlight, would result in a high incidence of osteoporosis and osteopaenia in this population. The results of the study indicate that over two-thirds of the study women had QUI scores indicative of osteoporosis or osteopaenia. This concurs with results of previous studies in the Peshawar survey of a larger group of over 600 women aged over 40 years, which revealed that almost 50% had decreased bone mineral density (T-score <-1) (16).

One of the strengths of the present study was that a detailed dietary analysis was undertaken over a 12-month period, enabling the dietary factors that may contribute to bone health to be evaluated. In general, the macronutrient content of the diet met energy requirements and were broadly in line with the dietary recommendations (20), with 60% energy from carbohydrate, 12% from protein, and 27% from fat. An adequate calcium intake is essential for bone mineralization, and zinc forms an important structural component of bone. The zinc intake met the World Health Organization's (WHO's) recommendation of 6.5 mg per day. However, the results of this study revealed that dietary calcium intakes in these women fell far below the WHO's recommendation of 1,300 mg per day. There were some seasonal fluctuations in total energy intake, with more calories being consumed in the winter months. Analysis of the 24-hour recall data indicated that this was reported to be primarily due to an increase in the consumption of hot milky tea during the cooler months, leading to an increase in protein, calcium and zinc intakes. However, calcium intake remained significantly below the recommended amount.

**Table 4.** Quantitative ultrasound T-score values, serum 25(OH)D, and PTH levels in a subgroup of participants

Category	T-score		25(OH)D (ng/mL)		PTH (pg/mL)	
	Median	No.	Median (range)	No.	Median (range)	No.
T-score above −1	0.00	32	23.95 (18.10-29.44)	13	89.7 (43.2-99.9)	13
T-score between −1 and 2.5	-1.65	46	17.91 (14.26-22.42)	13	73.8 (23.5-169)	13
T-score below −2.5	-2.8	27	18.71 (14.0-25.76)	11	84.2 (21.5-122)	11

PTH=Parathyroid hormone

In the present study, calcium intake did not correlate significantly with the QUI scores. A number of epidemiological studies have also failed to show an association between higher intakes of calcium and increased bone density or a decreased incidence of osteoporotic fracture (21,22). However, it is likely that calcium intake during childhood and adolescence plays a significant role in ensuring the acquisition of optimal peak bone density within genetic limits (23).

Fasting blood samples taken from a sample of the participant pool were analyzed for 25(OH)D and PTH levels. It is widely accepted that concentrations of less than 20 ng/mL represent vitamin D deficiency (24). Only nine of the 37 women sampled in the study had 25(OH)D levels that were above this cut-off value, suggesting a high prevalence of 25(OH)D deficiency in this population. Very few foods naturally contain vitamin D; hence, vitamin D requirements are met largely by exposure to sun. The practice of wearing a full burqa when outdoors in commonplace in the Nahaqi community limits exposure to sunlight and is the most likely reason for the low vitamin D status. Vitamin D plays an important role in the absorption of calcium; therefore, the low vitamin D status in these women compounds the low dietary calcium intake.

The main limitation of this study is the relatively small sample-size, particularly of the blood analyses which was due to the high cost of these analyses. Despite this small sample, we have demonstrated a highly-significant relationship between 25(OH)D and the QUI T-scores which warrants further investigation. We have also demonstrated a low dietary calcium intake by postmenopausal women in this community, which, coupled with low vitamin D status, will result in a very low rate of calcium absorption. This study highlights the importance of improving the calcium intake and vitamin D status of women living in this rural region of Khyber Pakhtunkhawa. Young women should, therefore, be particularly targeted through community nutrition and health-education programmes because the benefits of optimal nutrition can have the greatest impact on bone mineralization in late teens and early 20s.

## ACKNOWLEDGEMENTS

The authors gratefully acknowledge the support of the Abaseen Foundation (UK-registered Charity No. 1095882) which was instrumental in facilitating the study.
